# Characterisation of Phenolic Compounds in South African Plum Fruits (*Prunus salicina* Lindl.) using HPLC Coupled with Diode-Array, Fluorescence, Mass Spectrometry and On-Line Antioxidant Detection

**DOI:** 10.3390/molecules18055072

**Published:** 2013-05-02

**Authors:** Alet Venter, Elizabeth Joubert, Dalene de Beer

**Affiliations:** 1 Department of Food Science, Stellenbosch University, Private Bag X1, Matieland (Stellenbosch) 7602, South Africa; E-Mail: aletventer4@gmail.com; 2 Post-Harvest and Wine Technology Division, Agricultural Research Council (ARC), Infruitec-Nietvoorbij, Private Bag X5026, Stellenbosch 7599, South Africa; E-Mail: joubertL@arc.agric.za

**Keywords:** diode-array detection, fluorescence detection, HPLC, mass spectrometry, method validation, on-line antioxidant activity, phenolic compound, plum fruit, *Prunus salicina* Lindl.

## Abstract

Phenolic compounds are abundant secondary metabolites in plums, with potential health benefits believed to be due to their antioxidant activity, amongst others. Phenolic characterisation of South African *Prunus salicina* Lindl. plums is necessary to fully evaluate their potential health benefits. An HPLC method using diode-array detection (DAD) for quantification of phenolic compounds was improved and fluorescence detection (FLD) was added for quantification of flavan-3-ols. Validation of the HPLC-DAD-FLD method showed its suitability for quantification of 18 phenolic compounds, including flavan-3-ols using FLD, and phenolic acids, anthocyanins and flavonols using DAD. The method was suitable for characterisation of the phenolic composition of 11 South African plum cultivars and selections, including various types with yellow and red skin and flesh. The method was used in conjunction with mass spectrometry (MS) to identify 24 phenolic compounds. Neochlorogenic acid and cyanidin-3-*O*-glucoside were the major compounds in most of the plums, while cyanidin-3-*O*-glucoside was absent in Sun Breeze plums with yellow skin and flesh. Post-column on-line coupling of the ABTS^•+^ scavenging assay with HPLC-DAD enabled qualitative evaluation of the relative contribution of individual phenolic compounds to the antioxidant activity. The flavan-3-ols, neochlorogenic acid and cyanidin-3-*O*-glucoside displayed the largest antioxidant response peaks.

## 1. Introduction

*Prunus salicina* Lindl. plums are cultivated mainly for the fresh fruit market, while *Prunus domestica* Lindl. plums are used for the production of prunes. Both species display great diversity in shape, size, taste and appearance of the fruits from different cultivars. In particular, various skin (yellow, red, dark purple) and flesh (yellow, red) colours occur. South Africa is one of the major producers of *P. salicina* plums and breeding programmes provide cultivars suited to the South African climate with a variety of commercially important properties. Plums and other fruit are considered important as part of a healthy diet, contributing vitamins, fibre and phenolic compounds. Phenolic compounds are of particular interest due to their contribution to maintaining optimal health [1]. However, it is necessary to gain more information regarding the identity, amount and antioxidant activity of phenolic compounds in plums.

The phenolic composition of both *P. salicina* [2,3,4] and *P. domestica* [5,6,7,8,9] plums has been investigated previously. Neochlorogenic acid and the anthocyanins cyanidin-3-*O*-glucoside or -rutinoside are reported as the main phenolic compounds in cultivars with red skin or flesh. No anthocyanins are present in cultivars with yellow skin and flesh [10]. Large qualitative and quantitative differences are also observed between cultivars and selections due to genetic differences [2,7]. The diversity in phenolic profiles places high demands on the analytical methods used for their identification and quantification, necessitating adjustment of methods for specific purposes. Several aspects of an HPLC method that can be adapted to improve the separation of target compounds, include the stationary phase (column), mobile phase composition, gradient program and column temperature. The use of different detectors may also be advisable for quantification of compounds from multiple phenolic groups. A diode array detector (DAD) allows detection at a range of wavelengths in the UV and visible spectra simultaneously, allowing classification of compounds from their UV-Vis spectral characteristics and quantification of different phenolic groups at appropriate wavelengths. Fluorescence detection (FLD), on the other hand, can be used to increase sensitivity for compounds with fluorescent properties, but low UV-Vis absorption, e.g., flavan-3-ols. Mass spectrometric (MS) detection is used to identify compounds based on their molecular weights and fragmentation patterns.

Coupling of HPLC analysis with antioxidant assays (known as on-line antioxidant assays) can be achieved through the post-column reaction of the antioxidant assay reagents with the HPLC effluent. The second chromatogram indicating antioxidant activity is then aligned with the corresponding compounds in the DAD chromatogram to identify individual phenolic compounds exhibiting antioxidant activity [11].

In-depth characterisation of the phenolic composition of South African *P. salicina* plums has not been reported to date. Only one publication reported a limited investigation of the major phenolic compounds in one cultivar [12]. The aim of this study was therefore to improve the HPLC method to separate effectively a larger number of individual phenolic compounds present in South African plums. Eleven cultivars and selections with diverse skin and flesh colours were chosen (Table 1) to ensure general suitability of the method for qualitative and quantitative analyses of South African plums. The method was validated to ensure its applicability for quantification and subsequently used to identify phenolic compounds in the different plum cultivars and selections using HPLC-DAD-MS. The method was coupled to an on-line antioxidant assay to evaluate the relative contribution of individual phenolic compounds to the antioxidant activity.

**Table 1:** Evaluated South African plum (*Prunus salicina* Lindl.) selections and cultivars.

Cultivars/selections	Skin colour (ripe)	Flesh Colour (ripe)	Harvest date
Sun Breeze	Yellow	Yellow	14 February 2012
Laetitia	Red	Yellow	8 February 2012
African Delight	Red	Yellow	12 February 2012
Sapphire	Red	Yellow	13 December 2011
Ruby Red	Red	Red	3 January 2012
Ruby Crunch (PR02-62) ^a^	Red	Red	31 January 2012
PR02-55	Red	Red	21 December 2010
PR03-34	Red	Red	20 December 2011
PR04-19	Red	Red	13 December 2011
PR04-32	Red	Red	17 January 2012
PR04-35	Red	Red	20 December 2011

^a^ Selection PR02-62 was released as a cultivar, Ruby Crunch, in May 2012.

## 2. Results and Discussion

### 2.1. High Performance Liquid Chromatography with Diode-Array and Fluorescence Detection (HPLC-DAD-FLD) Method Optimisation

The HPLC method earlier used for quantification of the major phenolic compounds (five compounds only) in a South African red-fleshed plum cultivar [12] was deemed unsuitable for an in-depth study on the phenolic composition of a range of South African plum cultivars. Adjustments were necessary to improve separation in a few areas of the chromatogram to allow evaluation of a broader spectrum of compounds and support analysis of a range of cultivars and selections. Other methods used for quantification of phenolic compounds in *P. salicina* and *P. domestica* fruits were also not deemed suitable, due to either a low number of quantified compounds (<15 compounds) [2,4,6,8,9], long analysis times (>120 min) [7] and/or the use of mobile phases not compatible with MS analysis [2,8]. Earlier, South African plums were analysed using a Gemini-NX C18 column (150 × 4.6 mm, 3 µm particle size) with 7.5% formic acid in water (Solvent A) and 7.5% formic acid in acetonitrile (Solvent B) as mobile phases [12]. As the Gemini-NX C18 column provided satisfactory peak shapes, other stationary phases were not investigated. The mobile phases, gradient program and column temperature were adjusted systematically from the previously described method until the desired separation was achieved. The improved method used aqueous 0.05% trifluoroacetic acid (TFA) and acetonitrile as mobile phases at 40 °C with an adjusted gradient (total run time = 45 min). The TFA was selected due to the need for mobile phase pH < 2 to keep the anthocyanin compounds in their flavylium cation form (red colour) for analysis, while providing less corrosive conditions than 7.5% formic acid for the HPLC pump. Although suppression of ionisation was observed with the use of TFA, acceptable MS data could still be acquired. The addition of fluorescence detection allowed quantification of flavan-3-ol compounds, which was not possible with the method of De Beer *et al.* [12]. The improved method was deemed suitable for quantification of 18 compounds in 11 South African plum cultivars and selections based on validation results (Section 2.3) and visual inspection of chromatograms for all cultivars and selections.

### 2.2. Identification and Quantification of Phenolic Compounds

**Figure 1:**
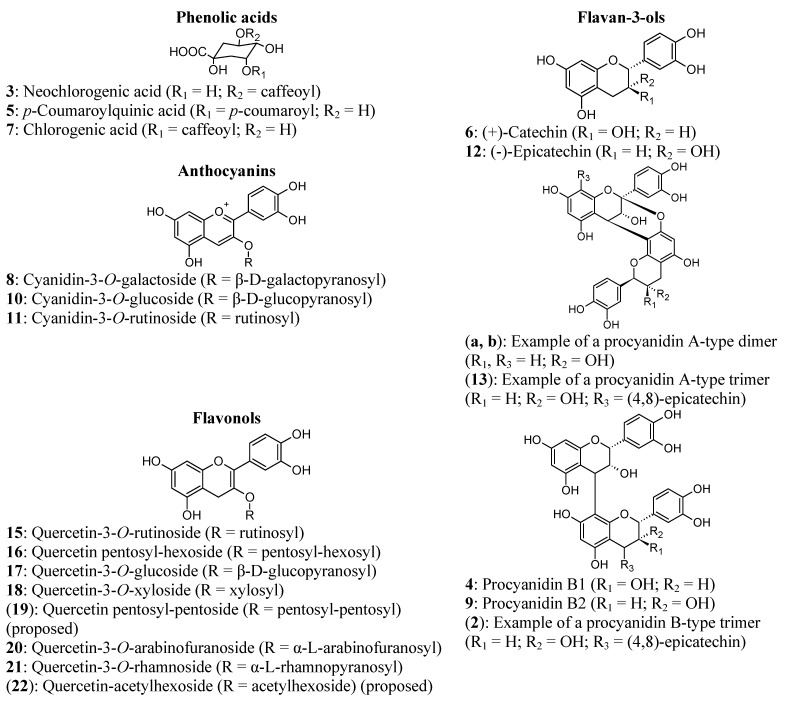
Structures of phenolic compounds present in South African plums (*Prunus salicina* Lindl.). Compounds **2**, **13**, **19**, **22**, **a** and **b** are not fully identified, but proposed structures or examples of possible structures are given.

Twenty three phenolic compounds from four phenolic groups, namely phenolic acids, anthocyanins, flavonols and flavan-3-ols, were identified or tentatively identified using HPLC-DAD-MS by comparing UV-Vis spectra, mass-to-charge ratio (*m/z*) values for the pseudo-molecular ion and compound fragments to that of literature and/or authentic standards (Table 2). Structures of the compounds, where known, are shown in Figure 1. During HPLC-DAD-FLD a number of compounds were identified by comparing their UV-Vis spectra and retention times to those of authentic reference standards. Their identities were also confirmed by HPLC-DAD-MS. Additional peaks were identified or tentatively identified from their pseudo-molecular ions ([M+H]^+^ or [M]^+^) and MS/MS fragments.

**Table 2:** UV-Vis and mass spectrometric characteristics of phenolic compounds identified in South African plums (*Prunus salicina* Lindl.).

Peak no.	t_R_ (min) ^a^	λ_max_ (nm)	[M+H]^+^/ M^+ b^	Na-adduct ions	Fragment ions	Identification
**1**	5.5	275	353	-	177, 160 *	Unknown compound 1
**2**	6.6	279	867	-	579 *, 247	B-type procyanidin trimer 1
**3**	8.4	324	355	-	163	Neochlorogenic acid ^c^
**4**	10.3	278	579	-	427, 409 *, 291	Procyanidin B1 ^c^
**5**	10.6	312	339	-	147	3 *-O-p*-Coumaroylquinic acid
**6**	10.8	278	291	-	139	(+)-Catechin ^c^
**7**	11.8	325	355	-	163	Chlorogenic acid ^c^
**8**	12.4	276, 515	449	-	287	Cyanidin-3 *-O-*galactoside ^c^
**9**	12.7	278	579	-	-	Procyanidin B2 ^c^
**10**	12.9	276, 515	449	-	287	Cyanidin-3 *-O-*glucoside ^c^
**11**	13.5	280, 515	595	-	287	Cyanidin-3 *-O-*rutinoside ^c^
**12**	13.3	278	291	-	139	(-)-Epicatechin ^c^
**13**	14.8	278	865	-	577, 425 *, 287	A-type procyanidin trimer
**14**	15.3	279	867	-	579 *, 409, 291, 247	B-type procyanidin trimer 2
**a ^d^**	16.8	278	577	-	425, 287 *	A-type procyanidin dimer 1
**15**	18.5	254, 352	611	633	465, 303 *, 229	Quercetin-3 *-O-*rutinoside ^c^
**16**	18.9	254, 351	597	619	303 *, 229	Quercetin pentosyl-hexoside
**17**	19.1	254, 353	465	-	303 *	Quercetin-3 *-O-*glucoside ^c^
**b ^d^**	19.1	278	577	-	425, 287 *	A-type procyanidin dimer 2
**18**	19.9	254, 351	435	891, 457	303 *, 229	Quercetin-3 *-O-*xyloside
**19**	20.1	254, 351	567	589	303 *, 229	Quercetin pentosyl-pentoside
**20**	20.6	255, 352	435	891, 457	303 *, 229	Quercetin-3 *-O-*arabinoside ^c^
**21**	21.1	255, 347	449	919, 471	303 *, 229	Quercetin-3 *-O-*rhamnoside ^c^
**22**	21.6	256, 351	507	529	303 *, 229	Quercetin-acetylhexoside

^a^ equivalent retention times for HPLC-DAD-FLD analysis using the Agilent 1200 HPLC to facilitate comparison with chromatograms; ^b^ M^+^ for anthocyanins and [M+H]^+^ for other compounds; ^c^ identified based on comparison to authentic reference standard; ^d^ no visible peaks on chromatograms; * ion with highest relative intensity.

In the phenolic acid group, neochlorogenic acid (3-*O*-caffeoylquinic acid, **3**) and chlorogenic acid (5-*O*-caffeoylquinic acid, **7**) were identified using authentic reference standards (Table 2). Both compounds had the same [M+H]^+^ (*m/z* 355) and fragment (*m/z* 163) ions. Generally, neochlorogenic acid is the predominant phenolic acid in plums, whether *P. salicina* or *P. domestica* [4,13,14,15]. The same was the case for South African plums in the current study, although this compound was absent in the cultivar Sapphire (Table 3).

**Table 3:** Phenolic composition (mg/kg fresh weight) of South African plum (*Prunus salicina* Lindl.) cultivars and selections.

Com-pound	Sun Breeze	Laetitia	African Delight	Sapphire	Ruby Red	Ruby Crunch	PR02-55	PR03-34	PR04-19	PR04-32	PR04-35
*Phenolic acids*											
**3**	218.0	366.6	391.1	nd	400.6	59.5	214.7	243.4	9.6	83.4	260.9
**5**	11.1	14.5	nd	nd	22.7	nd	94.8	111.0	nd	nd	108.0
**7**	nd	nd	24.7	nd	nd	nd	nd	11.3	nd	nd	21.6
*Anthocyanins*											
**8**	nd	4.3	nd	nd	nd	nd	nd	nd	nd	nd	nd
**10**	nd	47.9	82.1	222.6	456.8	221.3	38.3	238.0	461.8	84.6	190.9
**11**	nd	14.8	15.1	53.2	78.8	97.8	20.2	nq	145.2	31.8	114.4
*Flavonols*											
**15**	nq	65.5	73.9	23.1	28.1	79.5	5.1	14.7	22.1	9.9	14.3
**16**	5.6	5.3	9.7	8.7	nd	4.3	nd	6.0	10.6	nd	nd
**17**	19.2	69.3	143.3	62.8	88.9	85.5	5.6	27.7	49.5	14.8	18.0
**18**	4.8	5.2	2.7	6.5	8.5	4.2	3.0	3.7	6.2	3.0	5.8
**19**	2.5	1.9	2.1	4.0	3.7	nq	2.4	3.0	1.3	3.8	3.3
**20**	18.7	32.8	12.7	42.4	48.2	23.8	21.3	22.3	38.9	20.2	36.9
**21**	9.7	9.9	3.5	8.7	16.2	6.7	5.6	5.4	9.4	4.0	12.2
**22**	2.1	24.8	20.5	13.4	13.2	2.8	4.8	5.7	11.4	4.6	5.8
*Flavan-3-ols*											
**4**	203.7	100.8	119.8	87.1	62.5	271.4	189.6	200.7	24.3	218.2	318.4
**6**	65.6	61.5	65.5	71.3	53.9	171.5	106.2	114.0	33.6	172.0	177.0
**9**	16.1	7.6	12.4	53.6	50.0	32.7	13.4	14.5	18.9	101.8	46.7
**12**	8.3	10.2	6.3	58.0	54.6	41.3	14.8	20.8	42.3	93.2	36.3

Peak numbers as in Table 2. *Abbreviations*: nd, not detected; nq, not quantified due to low concentration or co-elution.

**Figure 2:**
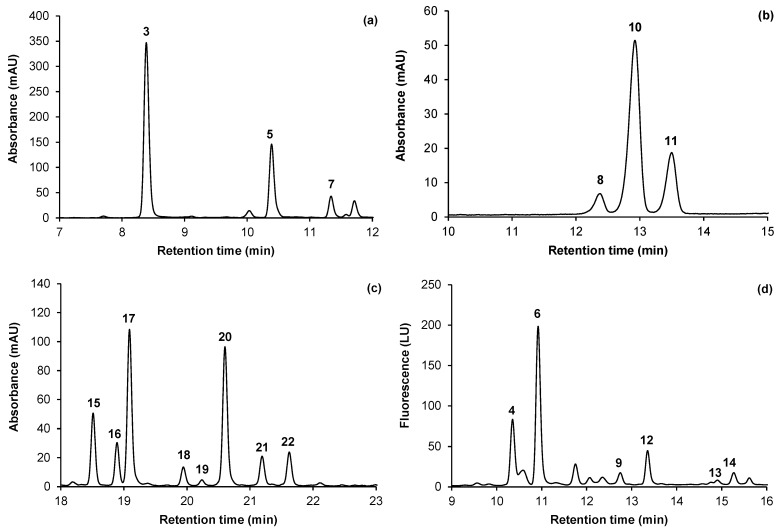
Enlarged sections of HPLC-DAD-FLD chromatograms for different phenolic groups in selected South African (*Prunus salicina* Lindl.) plum cultivars/selections (see Table 2 for peak numbers): (**a**) phenolic acids in PR04-35 at 320 nm; (**b**) anthocyanins in Laetitia at 520 nm; (**c**) flavonols in PR04-19 at 350 nm; (**d**) flavan-3-ols in Ruby Crunch using fluorescence detection.

In many cases, neochlorogenic acid was also the most abundant of all the quantified phenolic compounds, except in Ruby Red, Ruby Crunch, PR04-19, PR04-32 and PR04-35 [similar or lower content than cyanidin-3-*O*-glucoside (**8**)]. Chlorogenic acid has also been found in plums, although in lesser amounts than neochlorogenic acid [6,13]. A similar trend was observed for African Delight, PR03-34 and PR04-35, while chlorogenic acid was not detected in the other cultivars and selections. Compound **5**, displaying UV-Vis characteristics similar to that of chlorogenic and neochlorogenic acid, was detected in six out of the 11 plum cultivars and selections with contents lower than that of neochlorogenic acid (Table 3). The compound was identified as a *p*-coumaroylquinic acid from its [M+H]^+^ and fragment ions at *m/z* 339 and 147, respectively. The presence of a *p*-coumaroylquinic acid has previously been confirmed in plums [5,6,16], while Möller and Hermann [13] and Fang *et al.* [5] reported the presence of the 3-, 4- and 5-isomers of *p*-coumaroylquinic acid in stone fruit (including *P. domestica*). Based on their observation that the 3-isomers are the most abundant in stone fruit, compound **5** was tentatively identified as 3-*O-p*-coumaroylquinic acid. Figure 2a is an example of a chromatogram for selection PR04-35 at 320 nm showing the separation of the phenolic acid compounds.

The anthocyanins, cyanidin-3*-O-*galactoside (**8**), -glucoside (**10**) and *-*rutinoside (**11**), were identified using authentic reference standards (Table 2). The latter two compounds were present in all the plums except the cultivar Sun Breeze, with yellow skin and flesh (Table 3). Cyanidin-3-*O*-glucoside was present in much higher amounts in all cases. Laetitia was the only cultivar containing cyanidin-3*-O-*galactoside (Figure 2b), but at a very low concentration. Cyanidin-3*-O-*galactoside and -glucoside have the same [M]^+^ (*m/z* 449) and fragment (*m/z* 287) ions, while [M]^+^ and fragment ions at *m/z* 595 and 287, respectively, were observed for cyanidin-3-*O*-rutinoside. These anthocyanins were previously reported in *P. salicina* and *P. domestica* plums [4,6]. No peonidin glycosides (*i.e.*, peonidin-3-*O*-glucoside and -rutinoside), previously reported in *P. salicina* and *P. domestica* [2,6,8], were detected in any of the investigated South African plums.

Eight quercetin glycosides were detected in South African plums in the current study [Table 2; Figure 2(c)]. Quercetin-3*-O*-rutinoside (**15**), *-*glucoside (**17**), -arabinoside (**20**) and -rhamnoside (**21**) were identified using authentic standards. These compounds have previously been detected in plums [4,6,17]. Four other flavonol compounds, namely **16**, **18**, **19** and **22**, were present in the majority of the cultivars. From the HPLC-DAD-MS data these compounds were confirmed to be quercetin derivatives due to the presence of a fragment ion for the quercetin aglycone (*m/z* 303). Compound **18**, with [M+H]^+^ at *m/z* 435 and fragment ions at *m/z* 303 and 229, was tentatively identified as quercetin-3*-O-*xyloside. Quercetin-3*-O-*xyloside was distinguished from its arabinoside isomer during HPLC analysis by comparison of their retention times to that of an authentic standard for quercetin-3*-O-*arabinoside. Compounds **16** and **19** had [M+H]^+^ at *m/z* 597 and 567, respectively, and both showed fragment ions at *m/z* 303 and 229. They were identified as quercetin pentosyl-hexoside (**16**) and quercetin pentosyl-pentoside (**19**) based on previous tentative identification in *P. salicina* plum skin [4]. Compound **22** was tentatively identified as an quercetin-acetylhexoside, based on the [M+H]^+^ at *m/z* 507 and a loss of *m/z* 204 after fragmentation, matching the mass of a hexoside and acetyl residue (162 and 42 amu, respectively) [18]. Slimestad and Hostettmann [19] classified an unknown phenolic compound in Norwegian spruce with ions at *m/z* 507 and 303 as quercetin-3*-O-*(6-acetyl)glucoside, while small amounts of this compound were reported in *P. salicina* plums [4]. The relative abundance of the flavonol compounds differed between cultivars and selections, with quercetin-3-*O*-glucoside, quercetin-3-*O*-rutinoside and quercetin-3-*O*-arabinoside generally displaying higher contents that the other flavonol compounds (Table 3). All the flavonol compounds, except quercetin pentosyl-pentoside, were present in all the investigated cultivars and selections.

In the flavan-3-ol category the presence of the monomers, (+)-catechin (**6**) and (-)-epicatechin (**12**), as well as the B-type dimers, procyanidin B1 (**4**) and B2 (**9**), was confirmed using authentic reference standards (Table 2). Both monomers showed [M+H]^+^ ions at *m/z* 291 and fragment ions at *m/z* 139. The dimers had [M+H]^+^ ions at *m/z* 579, while fragment ions (*m/z* 427, 409 and 291) could only be detected for procyanidin B1. The presence of A-type procyanidin dimers (**a**, **b**) with [M+H]^+^ at *m/z* 577 was detected in some cultivars and selections, but peak numbers were not assigned as their corresponding peaks could not be distinguished on the chromatogram (Figure 2d). Due to the inter-catechin bonds of these compounds the pseudo-molecular ion has two mass units less than B-type procyanidin dimers [15]. The fragment ions also correspond to those of A-type procyanidins (*m/z* 425, 287) previously found in plums [4,15]. Other compounds were recognised as procyanidin B-type trimers (**2** and **14**) and an A-type trimer (**13**), with [M+H]^+^ corresponding to *m/z* 867 and 865, respectively, and fragment ions corresponding to the pseudo-molecular and fragment ions for the relevant dimers. Gu *et al.* [20] reported that the [M+H]^+^ at *m/z* 577 of the A-type trimer represents an interflavan bond between the middle and the base unit. However, the specific dimers and trimers could not be distinguished due to the lack of authentic reference standards. De Pascual-Teresa *et al.* [21] previously identified the B-type dimers procyanidin B3, B4, B5 and B7 and the B-type trimers procyanidin C1 and EC-(4,8)-EC-(4,8)-C in plums. Among the flavan-3-ols, only (+)-catechin, (-)-epicatechin, procyanidin B1 and procyanidin B2 could be quantified (Table 3). Procyanidin B1 was generally the most abundant flavan-3-ol compound followed by (+)-catechin, although large variation in relative abundance of the four compounds were observed among the cultivars and selections.

### 2.3. Method Validation

The HPLC-DAD-FLD method was validated by evaluating the linearity of calibration curves (Table 4), the compound stability in calibration mixtures and samples (Table 5) and intra- and inter-day precision in calibration mixtures and samples (Supplementary Information, Table S1). The linearity for authentic reference standards in the range expected in samples was excellent (*r^2^* > 0.999) and calibration curves had very small y-intercepts (Table 4). In terms of compound stability the % RSD values for compounds in the calibration mixtures were all below 5% (Table 5). Over the 28 h period the % change for the compounds in the calibration mixtures was between -5 and 5% in all cases, except for quercetin-3*-O-*rutinoside in the 1 µL injection (6.8%) and cyanidin-3*-O-*rutinoside in the 30 µL injection (−5.7%).

**Table 4:** Linear regression data for calibration curves.

Compound	Calibration range (µg injected on-column)	Slope	*Y*-Intercept	*r^2^*
Neochlorogenic	0.03–1.60	2482.8	−9.1	1.000
Chlorogenic acid	0.03–1.52	2680.0	−16.6	1.000
Cyanidin-3*-O-*glucoside	0.07–3.57	1889.1	6.9	1.000
Cyanidin-3*-O-*rutinoside	0.04–2.00	1918.4	8.1	1.000
Quercetin-3*-O-*rutinoside	0.01–0.51	1622.9	−1.4	1.000
Quercetin-3*-O-*glucoside	0.02–1.02	1692.1	−4.1	1.000
Quercetin-3*-O-*arabinoside	0.20–0.98	1854.6	−11.9	1.000
Quercetin-3*-O-*rhamnoside	0.02–0.99	1911.1	−7.5	1.000
(+)-Catechin	0.20–0.99	1444.6	3.4	1.000
(-)-Epicatechin	0.02–1.00	1121.7	1.6	1.000
Procyanidin B1	0.02–0.99	415.6	0.4	1.000
Procyanidin B2	0.02–1.00	750.3	−0.1	1.000

**Table 5:** Stability of compounds (%RSD and % change) present in calibration mixtures and two plum (*Prunus salicina* Lindl.) samples over a period of 28 h.

Compound	Calibration mixture (1 µL)		Calibration mixture (30 µL)		Ruby Red		African Delight	
	% RSD	% change	% RSD	% change	% RSD	% change	% RSD	% change
Neochlorogenic acid	1.1	2.3	0.1	0.1	0.3	0.8	0.3	1.1
Chlorogenic acid	1.0	0.0	0.1	0.4	nd	nd	1.3	2.8
3*-O-p*-Coumaroylquinic acid	n/a	n/a	n/a	n/a	1.4	−1.6	nd	nd
Cyanidin-3*-O-*glucoside	1.5	−1.4	1.4	−4.4	0.3	−0.7	0.8	−0.8
Cyanidin-3*-O-*rutinoside	2.4	−4.3	1.9	−5.7	0.7	−1.6	2.2	−1.3
Quercetin-3*-O-*rutinoside	2.7	6.8	0.1	0.1	0.2	0.2	0.3	1.1
Quercetin pentosyl-hexoside	n/a	n/a	n/a	n/a	nd	nd	2.1	1.9
Quercetin-3*-O-*glucoside	1.0	0.0	0.2	0.0	0.4	1.1	0.4	1.0
Quercetin-3*-O-*arabinoside	0.8	2.3	0.6	0.0	0.4	−1.1	0.5	1.1
Quercetin-3*-O-*rhamnoside	0.9	1.3	0.6	−0.1	0.3	0.8	1.5	2.5
Quercetin-3*-O-*xyloside	n/a	n/a	n/a	n/a	0.4	1.2	1.4	2.3
Quercetin pentosyl-pentoside	n/a	n/a	n/a	n/a	0.5	1.5	2.0	−2.0
Quercetin-acetylhexoside	n/a	n/a	n/a	n/a	0.3	0.7	0.3	0.6
(+)-Catechin	1.3	0.3	1.3	−0.6	1.1	2.6	1.1	2.1
(-)-Epicatechin	1.5	0.4	1.3	−0.7	1.5	2.0	2.0	3.0
Procyanidin B1	1.5	1.1	1.2	−0.9	1.4	2.8	1.2	3.4
Procyanidin B2	1.7	0.9	1.4	−0.9	2.3	0.9	2.0	3.1

*Abbreviations*: RSD, relative standard deviation; n/a, not applicable; nd, not detected.

The stability of compounds in the Ruby Red and African Delight reference samples was also excellent with % RSD less than 5% and % change between −5% and 5%. Excellent intra-day precision results (% RSD ≤ 4.1%) were obtained for all compounds in the calibration mixtures and the samples (Supplementary Information, Table S1). The inter-day precision (% RSD) for compounds in calibration mixtures and samples was excellent (<5%) in many cases, and acceptable (<10%) in most cases. The inter-day precision of (-)-epicatechin and procyanidin B2 in the African Delight sample was 11.6 and 13.2%, respectively. Poorer precision can be attributed to either small peak areas or difficulty integrating peaks due to interference from unidentified co-eluting compounds. In the case of the flavan-3-ol compounds, co-elution in samples is a problem warranting further improvement of the method.

### 2.4. On-line ABTS^•+^ Scavenging Antioxidant Assay

**Figure 3:**
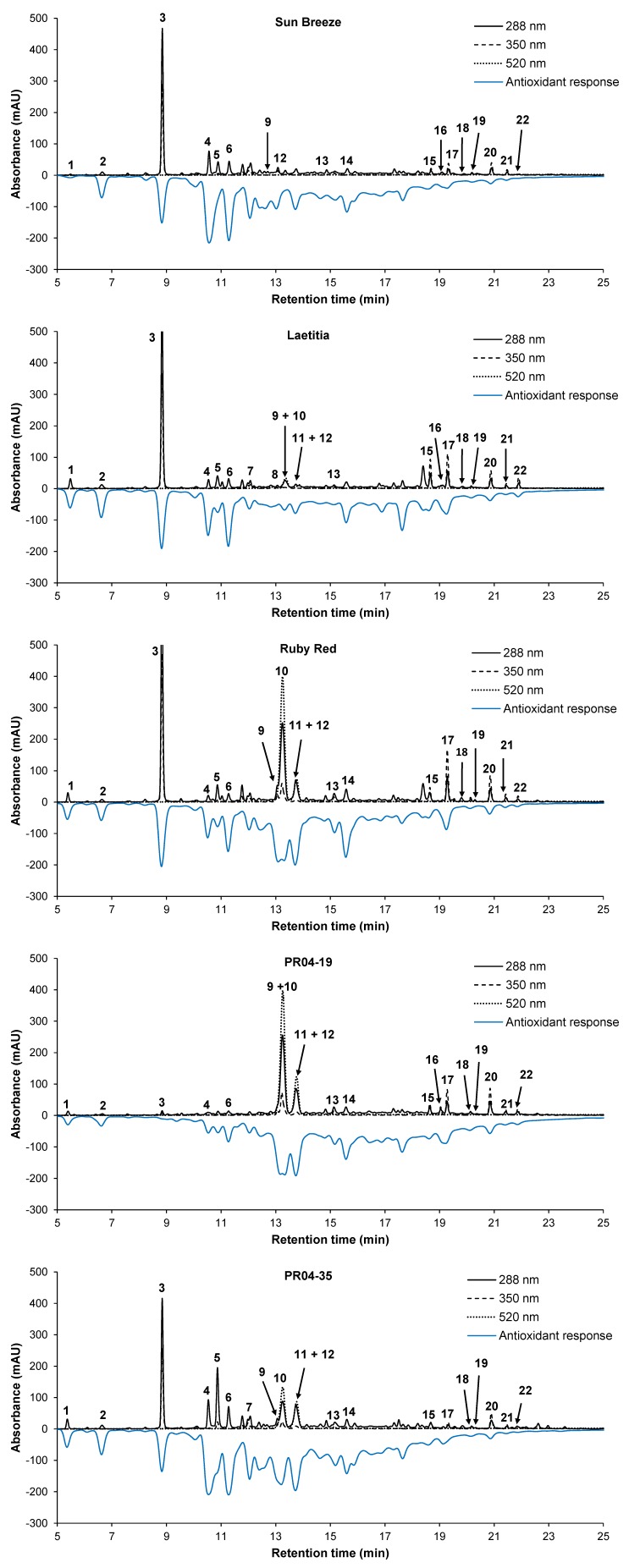
HPLC-DAD (positive peaks; black lines) and on-line antioxidant activity (negative peaks; blue line) chromatograms for selected South African plum (*Prunus salicina* Lindl.) cultivars and selections (peak numbers as in Table 2).

Eleven South African plum cultivars and selections were analysed using the on-line antioxidant assay (Figure 3; Supporting Information, Figure S1). The ABTS^•+^ on-line antioxidant assay is a useful analytical tool with one of the advantages being that the measured antioxidant activity is that of the individual compounds instead of a combined antioxidant activity as in the case of microplate assays [22]. The total antioxidant activity of a sample can also be affected by non-phenolic antioxidants and synergism between phenolic compounds. Post-column detection can be used to determine the antioxidant activity of individual compounds quantitatively where all phenolic compounds are well-separated from each other. During the current study this was not possible due to co-eluting peaks, resulting in broad co-eluted antioxidant response peaks, and unresolved oligomeric flavan-3-ols [23], resulting in an irregular antioxidant response baseline. Therefore, only a qualitative evaluation of the compounds important to the antioxidant activity was performed. The antioxidant response peaks for each compound indicate their relative contribution to the antioxidant activity of the sample with both potency and concentration taken into account.

The majority of the South African plum cultivars and selections showed a large antioxidant response peak corresponding to neochlorogenic acid, indicating that this compound has a large contribution to the antioxidant activity of the sample. Generally, the greatest antioxidant responses in each chromatogram corresponded to those of the flavan-3-ol compounds and in some cultivars, the anthocyanins.

Among the phenolic acids, neochlorogenic acid (**3**) mostly displayed the greatest antioxidant response as it was generally present in higher amounts. Selections PR03-34 and PR04-35 contained the highest 3-*O-p*-coumaroylquinic acid (**5**) content with a fairly large antioxidant response peak. In both cases, co-elution with procyanidin B1 (**4**) limited interpretation. In PR04-35, it seems that the response peak for 3-*O-p*-coumaroylquinic acid (**5**) and chlorogenic acid (**7**) is similar, despite a lower concentration of chlorogenic acid (**7**), indicating that chlorogenic acid may have higher antioxidant activity.

Both anthocyanins, namely cyanidin-3-*O*-glucoside (**10**) and -rutinoside (**11**), displayed large antioxidant response peaks in all samples except Laetitia (low anthocyanin content) and Sun Breeze (no anthocyanins). The antioxidant responses for the two anthocyanins were very similar despite the fact that cyanidin-3-*O*-glucoside was present in higher concentrations than cyanidin-3-*O*-rutinoside. No evaluation of their relative antioxidant activity was, however, possible due to co-elution of cyanidin-3-*O*-glucoside (**10**) with procyanidin B2 (**9**) and cyanidin-3-*O*-rutinoside (**11**) with (-)-epicatechin (**12**), leading to a combined antioxidant response or a split antioxidant response peak. An antioxidant response peak was not visible for cyanidin-3-*O*-galactoside in Laetitia due to its low concentration and co-elution with unidentified compounds. In plums with red skins the anthocyanins were previously shown to provide the greatest contribution to the total antioxidant activity (as determined by the ABTS^•+^ scavenging assay) among the major monomeric phenolic compounds [24].

Large antioxidant response peaks were generally observed for the flavan-3-ols. Procyanidin B1 (**4**) and (+)-catechin (**6**) showed a large antioxidant response for all cultivars and selections, with the exception of PR04-19, which had a low content of these compounds. In some cultivars the antioxidant response of procyanidin B1 (**4**) combined with that of 3-*O-p*-coumaroylquinic acid (**5**), due to close retention times. Tentatively identified B-type procyanidin trimers (compounds **2** and **14**) were also observed to display a relatively large antioxidant response compared to their UV-Vis peak areas. The UV-Vis peaks for these compounds at 288 nm are very small, yet a large antioxidant peak can be seen. One should keep in mind that the UV-Vis peaks are not an accurate representation of the flavan-3-ol concentration relative to the other phenolic compounds, as these compounds generally display low extinction coefficients in UV-Vis. In the chromatogram at 288 nm a slight but visible baseline drift is observed, which is likely due to the presence of unresolved oligomeric procyanidins [23]. This results in an uneven baseline in the antioxidant chromatogram caused by the combined antioxidant response of these compounds. From the size of the baseline distortion in the antioxidant response chromatogram these compounds should contribute greatly to the antioxidant activity of the samples.

The uneven baseline, however, prevented the separate evaluation of the antioxidant activity of the flavonol compounds especially, due to their low concentrations. Where antioxidant response peaks corresponding to flavonols could be distinguished, quercetin-3-*O*-glucoside (**17**) displayed the largest antioxidant activity of all the quercetin derivatives (Laetitia, African Delight, Ruby Red and Ruby Crunch), followed by either quercetin-3-*O*-rutinoside (**15**) or -arabinoside (**20**). These compounds were generally present in higher concentrations than the other flavonol compounds.

Compound **1**, present in all plum cultivars and selections investigated, also showed a fairly large antioxidant response peak. However, this compound could not be identified from its UV-Vis and MS characteristics.

## 3. Experimental

### 3.1. Chemicals

Authentic reference standards (purity indicated in brackets) were obtained from Fluka [Sigma-Aldrich, St. Louis, MO, USA; quercetin-3*-O-*glucoside (≥90%), quercetin-3*-O-*rhamnoside (≥97%) and chlorogenic acid (≥95%)], PhytoLab [Vestenbergsgreuth, Germany; quercetin-3*-O-*arabinoside (87%), neochlorogenic acid (98%), procyanidins B1 (94%) and B2 (95%)], Sigma-Aldrich [St. Louis, MO, USA; quercetin-3*-O-*rutinoside (≥94%), (+)-catechin (≥98%), (-)-epicatechin (≥98%)], Extrasynthese [Genay, France; cyanidin-3*-O-*rutinoside (97%)] and Polyphenols Laboratories [Sandnes, Norway; cyanidin-3*-O-*glucoside (≥97%) and -galactoside (≥97%)]. Trifluoroacetic acid (TFA) and acetonitrile (gradient grade for liquid chromatography) were purchased from Sigma-Aldrich. Formic acid and methanol (UniVar) were purchased from Merck Millipore (Darmstadt, Germany). The ABTS [2,2'-azinobis-(3-ethyl-benzothiazoline-6-sulphonic acid)] reagent was supplied by Roche Diagnostics GmbH [Indianapolis, IN, USA]. Laboratory grade deionised water was prepared using an Elix (Merck Millipore) water purification system and subsequently subjected to an additional purification step using a Milli-Q academic (Merck Millipore) water purification system to obtain HPLC grade water.

### 3.2. Harvesting of Fruit and Sample Preparation

Plums were harvested at Bien Donné experimental farm (Groot Drakenstein, South Africa; S 33.84, E 18.98) during December 2010 and from December 2011 to February 2012 (Table 1). Harvest dates were determined by evaluation of firmness and total soluble solids parameters, as set out by the Agricultural Product Standards Act [25] for different cultivars. Fruit underwent a commercial cold storage regime (10 days at −0.5 °C, 9 days at 7.5 °C and 16 days at −0.5 °C), followed by 7 days of ripening at 10 °C [26]. Fruit (five plums per sample) were homogenised with 4 g/L sodium fluoride (1 mL for every 4 g fruit) and samples frozen in 50 mL centrifuge tubes at ca −20 °C until extraction. For extraction, the frozen plum pulp was defrosted at room temperature and ca 5.0 g weighed into a 50 mL screw-cap centrifuge tube and 10 mL methanol added. Thereafter the tubes were shaken, placed in a sonication bath (Branson 8510, Branson Ultrasonic Corporation, Danbury, CT, USA) for 10 min, and centrifuged to separate the solids from the liquid. Centrifugation was performed for 10 min at 8000 rpm (ca 6000 × g) using a Biofuge primo Centrifuge (Thermo Scientific, AEC-Amersham, Johannesburg, South Africa). The supernatant was filtered using a Millex-HV hydrophilic poly(vinylidene difluoride) (PVDF) 0.45 µm syringe-driven filter (Merck Millipore). Thereafter 300 µL aliquots of the filtrate were diluted with 1 mL deionised water and frozen at ca −20 °C until HPLC-DAD-FLD analysis. For HPLC-DAD-MS and on-line antioxidant analyses, the filtrate (ca 12 mL) was concentrated to ca 2 mL using a Savant SPD 2010 SpeedVac Concentrator (Savant SPD 2010, Thermo Scientific, Waltham, MA, USA).

### 3.3. High-Performance Liquid Chromatography with Diode-Array and Fluorescence Detection (HPLC-DAD-FLD)

Analyses were performed using an Agilent 1200 series HPLC (Waldbronn, Germany). The system consisted of an autosampler, quaternary pump, column thermostat, diode-array detector and fluorescence detector. Chemstation software for LC 3D systems (Agilent) was used for data acquisition and analysis. A Gemini-NX C18 column (3 μm; 110 Å; 150 × 4.6 mm; Phenomenex, Santa Clara, CA, USA) protected by a guard column packed with the same stationary phase (4 × 3.0 mm; Phenomenex) was used. The method used by De Beer *et al.* [12] was modified to obtain improved separation of the phenolic compounds. The final mobile phases were 0.05% TFA (A) and acetonitrile (B). Analysis was performed at 40 °C and a flow rate of 1 mL/min. The mobile phase gradient was as follows: 0–2 min (3% B), 2–30 min (3–35% B), 30–31 min (35–50% B), 31–33 min (50% B), 33–35 min (50–3% B), 35–45 min (3% B). Hydroxycinnamic acids were quantified at 320 nm, flavonols at 350 nm and anthocyanins at 520 nm. Flavan-3-ols were quantified using a fluorescence detector (excitation = 275 nm; emission = 315 nm). Two injection volumes (100 µL and either 40 or 50 µL) were used for samples to ensure accurate quantification of compounds present in small concentrations and those present in large amounts. A calibration mixture containing the authentic reference standards was injected at 1, 5, 10, 20, 30, 40 and 50 µL to obtain calibration ranges as follows: neochlorogenic acid (0.03-1.6 µg injected); chlorogenic acid (0.03–1.5 µg); cyanidin-3*-O-*glucoside (0.07–3.2 µg); cyanidin-3*-O-*rutinoside (0.04–2.0 µg); quercetin-3*-O-*rutinoside and -galactoside (0.01–0.5 µg); quercetin-3*-O-*glucoside, -rhamnoside and -arabinoside (0.02–1.0 µg); (+)-catechin, (-)-epicatechin, procyanidin B1 and procyanidin B2 (0.02–1.0 µg). The calibration ranges were chosen to span the variation in the phenolic compound contents of the different cultivars and selections, as determined through trials during the development process.

### 3.4. Liquid Chromatography-Mass Spectrometry (HPLC-DAD-MS)

HPLC-DAD-MS analyses were performed using a Waters Synapt G2 QTOF mass spectrometer (Waters, Milford, MA, USA) with an electrospray ionisation (ESI) source. A Waters Acquity ultra-high-pressure liquid chromatography (UPLC) system equipped with autosampler, binary pump and DAD was used to perform separation using the same method parameters as for HPLC-DAD-FLD, except that an external column heating compartment was used. Concentrated plum extracts were injected (10 µL) and the HPLC effluent split 60:40 before introduction to the ionisation source. MS data were acquired in positive ionisation mode. MS^E^ mode was used to conduct both MS and automated MS/MS measurements during the same run. Collision energy was ramped from 15 to 60 V. The MS parameters were as follows: desolvation temperature, 275 °C; source temperature, 120 °C; nitrogen flow rate, 650 L/h; capillary voltage, 3 kV; cone voltage, 15 V. Data were acquired and processed using MassLynx v.4.1 software (Waters).

### 3.5. HPLC Method Validation

Seven-point calibration curves were set up for all of the standards (calibration mixture prepared identical to that used for HPLC quantification) in order to test the linearity of the DAD and FLD responses. Linear regression on the calibration curve data for each compound was performed using the least squares method (Microsoft Excel 2003, Microsoft Corporation, Redmond, WA, USA) and evaluated using the slope, y-intercept and coefficients of determination (*r^2^*).

Two representative plum samples (Ruby Red and African Delight) and a standard calibration mixture were injected over a 28.2 hour period to evaluate sample stability. The standard calibration mixtures were prepared in the same manner as for HPLC analysis and injected at 1 µL and 30 µL and the plum samples injected at 100 µL. The relative standard deviation (RSD) over all injections and percentage change over the period were determined for each compound. The inter- and intra-day precision of the method were evaluated by injecting the samples and standard mixtures six times each day for three consecutive days. The same injection volumes were used as for stability analysis. The RSD for each compound in the samples and standards was calculated for the replicate injections per day, as well as for the mean values of the three days.

### 3.6. On-line Antioxidant Analysis

The method of Pellegrini *et al.* [11] was modified for the on-line ABTS^•+^ antioxidant assay. The ABTS^•+^ stock solution was prepared according to Pellegrini *et al.* [27] by incubating ABTS and potassium-persulphate at room temperature for 12–16 h. The final working solution (25 mL ABTS^•+^ stock added to 1 L 75 mM potassium phosphate buffer at pH 7.4) was filtered before analysis and placed in a cooling unit (4 °C) to stabilise the radical for the duration of the analysis. The Agilent HPLC system used for quantification of phenolic compounds was coupled to additional components to perform the on-line antioxidant activity assay. An in-line X-Act degasser (Jour Research, Onsala, Sweden) was used to degas the ABTS^•+^ reagent. The HPLC effluent flow was mixed with ABTS^•+^ reagent delivered at 0.5 mL/min by a second pump (LKB Bromma 2150, Bromma, Sweden) using a high-pressure static mixing tee. The combined reagents flowed through a reaction coil (15.24 m PEEK tubing) which allows time (36 s) for scavenging of ABTS^•+^ by the separated compounds. The decrease in absorbance caused by the reaction was detected by a variable wavelength detector (Agilent 1200 series) at 600 nm. Trolox (40 µL; 1.25 mg/µL Trolox dissolved in ethanol) was added to 1 mL of concentrated plum extract as internal standard. The mixture was injected at 5 µL. Trolox served as reference to align the antioxidant response peaks with the corresponding phenolic compound peaks on the DAD chromatogram.

## 4. Conclusions

Plums, especially red-fleshed cultivars, are of interest for their health-promoting properties imparted by a generally high concentration of phenolic compounds. In-depth evaluation of their phenolic composition and antioxidant capacity is important for their evaluation as healthy fruit. During the current study a previous HPLC method developed by our group for quantification of the 5 major phenolic compounds in plums was greatly improved, allowing quantification of 18 phenolic compounds. Compounds from four phenolic groups, namely phenolic acids, anthocyanins, flavonol and flavan-3-ols, were successfully identified. However, where the quantification of flavan-3-ol compounds is of primary interest, further optimisation of the method is needed. Results clearly display large variation in the phenolic profiles of 11 South African plum cultivars and selections. The ABTS^•+^ on-line antioxidant assay revealed that many of the phenolic compounds, mainly from the phenolic acid, anthocyanin and flavan-3-ol classes, contribute greatly to the total antioxidant activity of the fruit.

This is the first study to provide comprehensive characterisation of the phenolic profile of South African plum cultivars and selections. It is believed that the optimised HPLC method and knowledge about the specific phenolic compounds present will be a helpful tool for future research on South African plums, especially in breeding programs and the evaluation of new plum selections. Future work will entail a quantitative investigation of differences in phenolic composition and antioxidant activity of a number of cultivars and selections over two harvest seasons.

## Supplementary Materials

Supplementary materials can be accessed at: http://www.mdpi.com/1420-3049/18/5/5072/s1.
