# Supplementary material for: Characterisation of Phenolic Compounds in South African Plum Fruits (Prunus salicina Lindl.) using HPLC Coupled with Diode-Array, Fluorescence, Mass Spectrometry and On-Line Antioxidant Detection
Source: Molecules. 2013 May 2;18(5):5072–90. doi: 10.3390/molecules18055072 (PMC6270170; doi:10.3390/molecules18055072)

Supplementary Materials

Table S1. Intra- and inter-day precision (% RSD) for selected phenolic compounds in calibration mixtures and two plum (*Prunus salicina* Lindl.) samples.

Sample	Compound	Day 1 (n = 6)	Day 2 (n = 6)	Day 3 (n = 6)	Inter-day (n = 3)
Calibration mixture (1 µL) [a]	Neochlorogenic acid	2.1	0.6	0.7	3.0
	Chlorogenic acid	1.6	0.8	0.6	2.4
	Cyanidin-3-*O*-glucoside	2.2	1.9	1.7	4.2
	Cyanidin-3-*O*-rutinoside	2.4	1.5	1.5	3.8
	Quercetin-3-*O*-rutinoside	2.0	1.2	2.3	5.7
	Quercetin-3-*O*-glucoside	1.2	1.0	3.0	2.7
	Quercetin-3-*O*-arabinoside	1.4	0.5	1.3	3.5
	Quercetin-3-*O*-rhamnoside	1.2	2.3	1.8	5.3
	(+)-Catechin	2.1	3.1	0.9	7.1
	(-)-Epicatechin	2.3	3.5	0.8	6.4
	Procyanidin B1	2.5	3.2	1.4	1.2
	Procyanidin B2	2.5	3.6	0.9	6.6
Calibration mixture (30 µL)[a]	Neochlorogenic acid	0.2	0.1	0.2	0.3
	Chlorogenic acid	0.2	0.1	0.2	2.3
	Cyanidin-3-*O*-glucoside	0.2	0.3	0.1	0.7
	Cyanidin-3-*O*-rutinoside	0.3	0.5	0.2	2.2
	Quercetin-3-*O*-rutinoside	0.1	0.1	0.1	7.5
	Quercetin-3-*O*-glucoside	0.2	0.2	0.2	6.1
	Quercetin-3-*O*-arabinoside	0.6	0.5	0.2	3.7
	Quercetin-3-*O*-rhamnoside	0.1	0.1	0.2	6.1
	(+)-Catechin	0.4	0.1	0.2	5.3
	(-)-Epicatechin	0.2	0.3	0.3	4.6
	Procyanidin B1	0.3	0.2	0.3	7.5
	Procyanidin B2	0.2	0.3	0.3	4.6
Ruby Red	Neochlorogenic acid	0.2	0.2	0.2	1.1
	Chlorogenic acid	nd	nd	nd	nd
	3-*O*-*p*-Coumaroylquinic acid	1.7	1.7	0.3	1.6
	Cyanidin-3-*O*-glucoside	0.1	0.0	0.1	2.2
	Cyanidin-3-*O*-rutinoside	0.3	0.1	0.2	1.4
	Quercetin-3-*O*-rutinoside	0.4	0.1	0.2	1.0
	Quercetin pentosyl-hexoside	nd	nd	nd	nd
	Quercetin-3-*O*-glucoside	0.1	0.1	0.1	1.5
	Quercetin-3-*O*-arabinoside	0.3	0.1	0.4	2.3
	Quercetin-3-*O*-rhamnoside	0.1	1.5	0.2	1.2
	Quercetin-3-*O*-xyloside	0.1	0.3	1.1	2.7
	Quercetin pentosyl-pentoside	0.5	0.4	3.1	3.2
	Quercetin-acetylhexoside	0.2	0.4	0.3	1.6
	(+)-Catechin	0.5	0.2	0.1	6.4
	(-)-Epicatechin	0.6	1.0	1.2	7.4
	Procyanidin B1	1.4	4.1	3.2	4.2
	Procyanidin B2	0.2	2.2	0.3	8.7

Table S1. *Cont.*

Sample	Compound	Day 1 (n = 6)	Day 2 (n = 6)	Day 3 (n = 6)	Inter-day (n = 3)
African Delight	Neochlorogenic acid	0.9	0.1	0.1	0.8
	Chlorogenic acid	1.2	0.5	1.0	2.9
	3-*O*-*p*-Coumaroylquinic acid	nd	nd	nd	nd
	Cyanidin-3-*O*-glucoside	1.0	0.4	0.6	1.5
	Cyanidin-3-*O*-rutinoside	2.6	2.1	2.2	1.1
	Quercetin-3-*O*-rutinoside	0.1	0.1	0.2	0.5
	Quercetin pentosyl-hexoside	0.3	2.0	0.2	2.9
	Quercetin-3-*O*-glucoside	0	0.2	0.2	1.0
	Quercetin-3-*O*-arabinoside	0.5	0.6	0.1	2.2
	Quercetin-3-*O*-rhamnoside	1.3	0.5	0.7	1.0
	Quercetin-3-*O*-xyloside	0.9	1.2	1.0	2.3
	Quercetin-pentosyl-pentoside	3.4	3.4	1.7	0.8
	Quercetin-acetylhexoside	0.2	0.4	0.3	1.6
	(+)-Catechin	0.9	0.4	0.7	7.4
	(-)-Epicatechin	0.7	0.7	1.2	11.6
	Procyanidin B1	1.4	0.9	1.3	9.6
	Procyanidin B2	0.7	0.6	0.8	13.2

[a] Neochlorogenic and chlorogenic acid at 0.03 mg/mL; cyanidin-3-*O*-glucoside at 0.07 mg/mL; cyanidin-3-*O*-rutinoside at 0.04 mg/mL; quercetin-3-*O*-rutinoside at 0.01 mg/mL; quercetin-3-*O*-glucoside, -arabinoside, -rhamnoside, (+)-catechin, (-)-epicatechin, procyanidin B1 and procyanidin B2 at 0.02 mg/mL. *Abbreviations:* RSD, relative standard deviation.

Figure S1. HPLC-DAD (positive peaks; black lines) and on-line antioxidant activity (negative peaks; blue line) chromatograms for South African plum (*Prunus salicina* Lindl.) cultivars and selections not shown in Figure 3 (peak numbers as in Table 2).

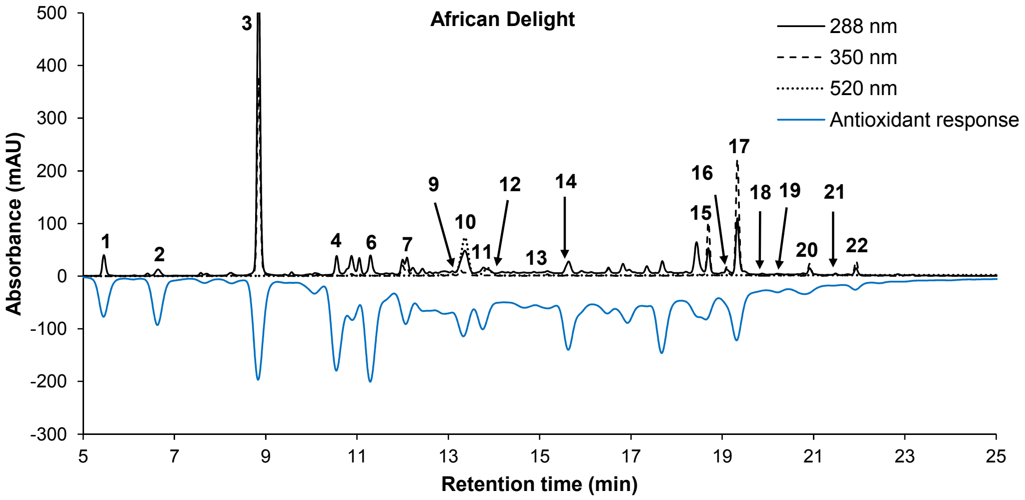

Figure S1. *Cont.*

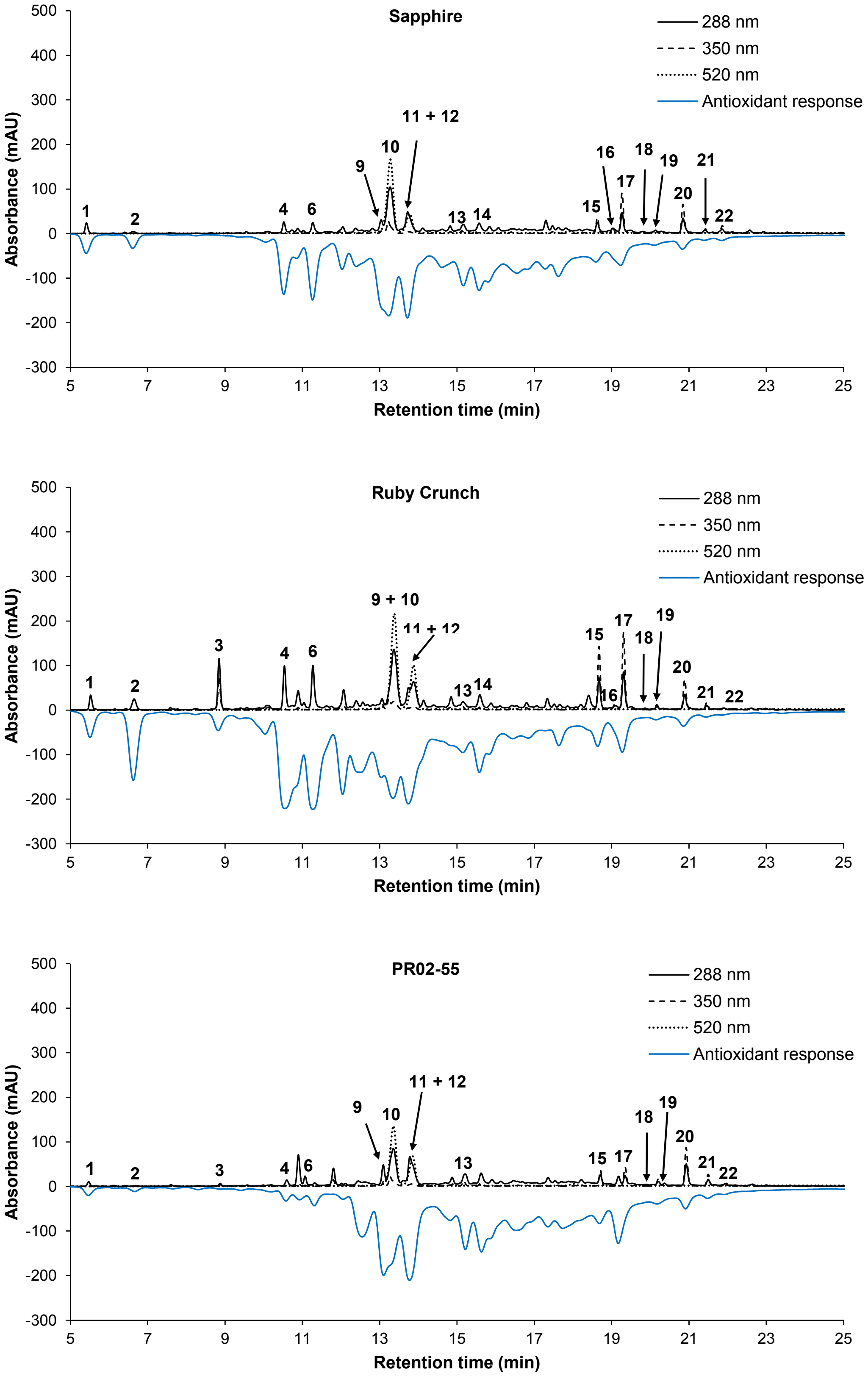

Figure S1. *Cont.*

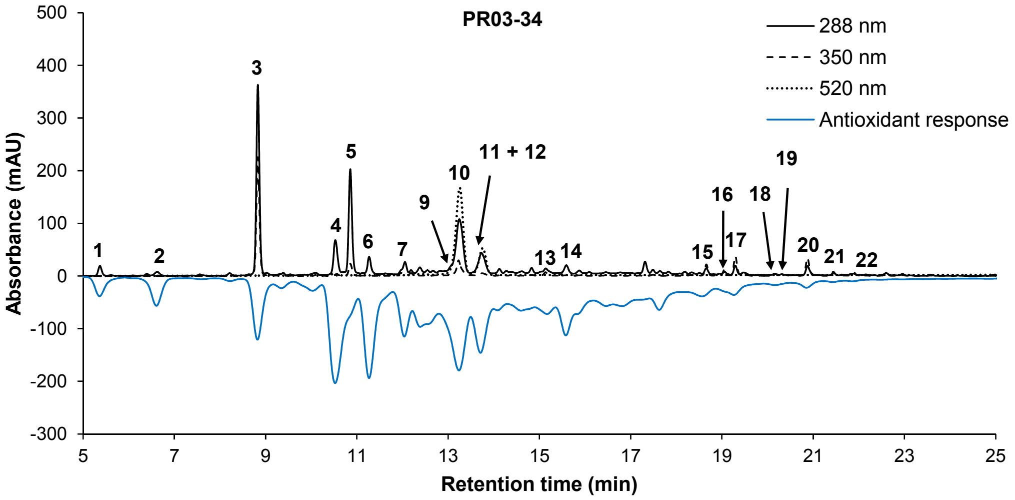

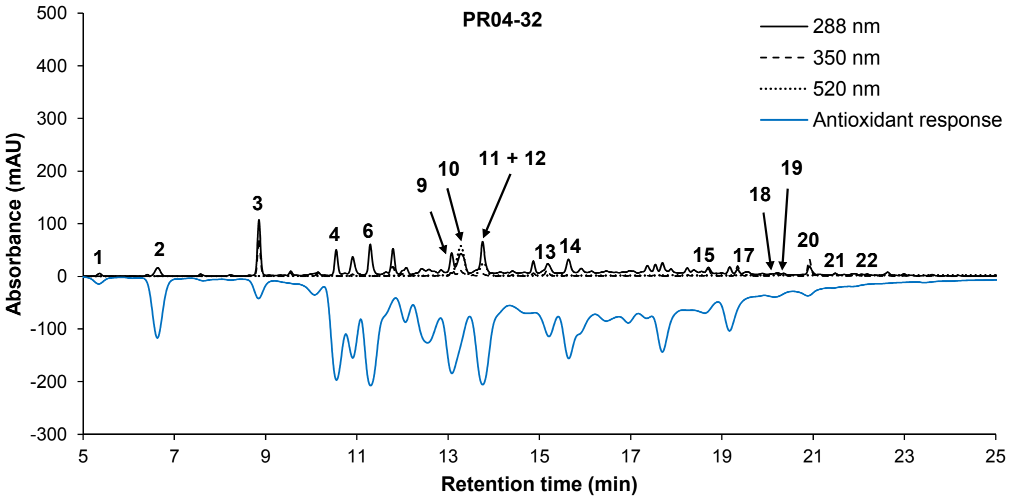

Supplement: Supplementary file 1 [file molecules-18-05072-s001.pdf]
